# Antidepressant use in suicides: a case-control study from the Friuli Venezia Giulia Region, Italy, 2005–2014

**DOI:** 10.1007/s00228-017-2236-0

**Published:** 2017-03-24

**Authors:** Giulio Castelpietra, Michele Gobbato, Francesca Valent, Clarissa De Vido, Matteo Balestrieri, Göran Isacsson

**Affiliations:** 1 Department of Clinical Neuroscience, Division of Psychiatry, Karolinska Institutet, Stockholm, Sweden; 2 Italian Collaborating Centre for the WHO Family of International Classifications, Udine, Italy; 3 Primary care services Area, Central Health Directorate, Region Friuli Venezia Giulia, Trieste, Italy; 4 Epidemiological Service, Central Health Directorate, Friuli Venezia Giulia Region, Udine, Italy; 5 Department of Medical Sciences, University of Trieste, Trieste, Italy; 6 Psychiatric Unit, Department of Experimental and Clinical Medical Sciences, University of Udine, Udine, Italy

**Keywords:** Suicide, Antidepressants, Adherence, Current use, Case-control

## Abstract

**Purpose:**

To compare the use of antidepressant (AD) classes and compounds in individuals who committed suicide and in controls from the general population and to assess to what extent adherence and current use of different AD classes can affect the risk of committing suicide.

**Methods:**

Individual data on suicide, diagnoses and AD use in Friuli Venezia Giulia from 2005 to 2014 were obtained from the Regional Social and Health Information System. All suicides that had at least one prescription of AD in the 730 days before death (*N* = 876) were included as cases. Each case was matched with regard to age and sex with five controls from the general population. The association between suicide and AD use was assessed using conditional logistic regression analysis.

**Results:**

Almost 70% of all suicides occurring in the10-year period had been prescribed AD. Selective serotonin reuptake inhibitors (SSRIs) accounted for more than the 90% of the prescriptions, with paroxetine the most prescribed AD. All AD compounds and classes were not associated with a higher suicide risk, with the exception of SSRI (OR = 1.6). A decreasing trend in suicide risk was observed when adherent subjects or current AD users were compared to the others.

**Conclusions:**

AD treatment is an important factor for preventing suicide, since the use of AD at adequate dosage and for a proper duration was associated with a lower suicide risk. The proper use of AD should be ascertained by physicians, particularly in a primary care context.

**Electronic supplementary material:**

The online version of this article (doi:10.1007/s00228-017-2236-0) contains supplementary material, which is available to authorized users.

## Introduction

Suicide is a major public health problem. There is a close link between suicide and psychiatric diseases, foremost depression [1]. Depression has repeatedly been found to be untreated in affected individuals [2, 3]. Several studies have documented a low adherence to antidepressant medication in the general population, both at the international [4–7] and at the Italian level [8, 9]. Early discontinuation of antidepressants among diagnosed patients has also been reported [6, 10–14]. Modifications, such as switching or combining different antidepressants, have been shown to seldom occur [9, 11, 14, 15]. In many countries, however, increased use of antidepressants during the last two decades has been accompanied by decreased suicide rates [16, 17]. Such an association has been documented also in the Italian region of Friuli Venezia Giulia (FVG) [3]. Furthermore, there is evidence from a Swedish individual-based study that the association may be the expression of a causal relationship, and that antidepressants actually prevent suicide [2]. A British study found that the risk of suicide among patients treated with antidepressants decreased with the length of treatment [13]. We had similar findings in FVG [18]. Furthermore, our study found that the risk of suicide in patients with somatic disorders was not higher than the risk of the general population, when patients were adherent to antidepressant medication [19]. Current use of antidepressants was also associated with a lower risk of suicide deaths [20, 21]. Nonetheless, there is a need for more studies of the effects of antidepressants on suicide risk, particularly with a focus on qualitative parameters.

The aims of the present case-control study are the following: (i) to compare the pattern of use of different antidepressant classes and compounds in individuals who committed suicide and in controls from the general population; and (ii) to assess to what extent adherence and current use of different antidepressant classes can affect the risk of committing suicide.

## Material and methods

### Study design, subjects and diagnoses

Cases and controls were selected from the Regional Social and Health Information System (SISSR) of the Friuli Venezia Giulia (FVG) Region, Italy. The system provides a unique anonymous key, which allows for the linking of data from different regional databases (the Death Register, the Hospital Discharge Register and the Drug Prescription Register) [19].

Subjects who committed suicide during a 10-year period (from 1 January 2005 to 31 December 2014) were identified through the Death Register, using ICD-9 codes E95* and E98* for intentional self-harm and events of undetermined intent. Suicides were then defined as cases provided that they also had received at least one prescription of an antidepressant during their last 2 years (730 days) of life. For each case, five controls were selected from the FVG general population by using an incidence density sampling method [22]. Controls were matched by gender and year of birth, had to be alive at the time of suicide of their corresponding case (index date) and had to have received at least one prescription of an antidepressant in the 730 days prior to the index date.

In the event that suicides and controls had been hospitalised during the 2 years prior to the index date, the main in-patient diagnoses were obtained from the Hospital Discharge Register. Diagnoses were recorded as ICD-9 codes in the first position on the medical record on discharge from public and private hospitals covered by the Regional Health System. As in our previous study [19], they were arranged into three groups: affective disorders (codes 296, 300.4, 311); non-affective psychiatric disorders (codes 290–295, 297–300.3, 300.5–310, 312–319); and somatic disorders (codes 001–289, 320–629, 680–759, 780–799). Complications from pregnancy, childbirth and puerperium (codes 630–679); certain conditions originating in the perinatal period (codes 760–779); injury and poisoning (codes 800–999) and external causes of injury and supplemental classification (codes E and V) are not considered and excluded from the data analyses. The time range for all the diagnostic variables is the 730 days prior to the index date.

### Antidepressant prescriptions

Antidepressant data includes all prescriptions filled in FVG in the 730 days prior to the index date, if prescribed by a general practitioner (GP) or other public physician. Such prescriptions are reimbursed by the National Health System and cover more than 90% of all antidepressant prescriptions in the region [3]. The retrieved data included the prescription date, the number of packages, the volume (expressed in defined daily doses, DDD), the specific antidepressant drug name and its ATC code [23].

The different antidepressant drugs were merged into four classes: tricyclics TCA (ATC code N06AA), selective serotonin reuptake inhibitors (SSRI) (N06AB), serotonergic noradrenergic reuptake inhibitors (SNRIs) (N06AX21, N06AX16) and “other” antidepressants (N06AX49, N06AX12, N06AX11, N06AF03, N06AX03, N06AX18, N06AX05). The tetracyclic maprotiline (ATC code N06AA2) was included in the TCA category.

The total number of prescriptions of each specific antidepressant as well as the number of each class was analysed.

Additionally, it determined whether a subject adhered to antidepressants or was currently using antidepressants at the time of the index date.

Adherence to antidepressants was assessed using the medication possession ratio (MPR), which is defined as the proportion of days supplied under a specified time period [24]. The total number of DDD approximated the number of days of treatment:

$$ \mathrm{MPR}=\frac{\mathrm{Total}\ \mathrm{number}\ \mathrm{of}\ \mathrm{DDD}\ \mathrm{in}\ \mathrm{the}\ 730\ \mathrm{days}\ \mathrm{prior}\ \mathrm{to}\ \mathrm{the}\ \mathrm{in}\mathrm{dex}\ \mathrm{date}}{730\ \mathrm{days}}\times 100 $$

MPR was divided into two groups (1–79; ≥80%). Only individuals with an MPR ≥ 80% were defined as adherent to treatment. Adherent individuals were covered by antidepressants for at least 584 days during the 730 days prior to the index date.

Current use of antidepressants at the time of the index date was determined by two conditions. First, the total number of DDD supplied at the second-to-last prescription was sufficient to cover the time up to the last prescription. Second, the total number of DDD supplied at the last prescription was sufficient to cover the time up to the index date.

Furthermore, we assessed the treatment modifications using two parameters:

1. The number of switches of antidepressant treatments. A switch is defined as the discontinuation of an index antidepressant and the prescription of another specific antidepressant. A delay up to 31 days until the prescription of the new antidepressant as well as an overlap of the two drugs up to 31 days will be allowed [11].
2. The number of combinations of antidepressant treatments. Combination is defined as the prescription of the index antidepressant overlapping the prescription of a second antidepressant for more than 31 days [11].

The numbers of switches and combinations were calculated with regard to all the antidepressant compounds, as well as to the four classes of antidepressants (i.e. TCA, SSRI, SNRI, other).

### Statistical analyses

Continuous variables were summarised using the median as a measure of central tendency and the range as a measure of dispersion, whereas dichotomous or categorical variables are tabulated into contingency tables. For categorical variables, the chi-square statistic (*χ*2) was used to test the differences between observed and expected frequencies.

Conditional logistic regression analysis was used to assess the associations between outcome (suicide) and predictors (use of antidepressant).

Crude and adjusted odds ratios (OR) and 95% confidence intervals (95% C.I.) were estimated from the logistic regression coefficients and their respective standard errors. A *P* value (*P*) < 0.05 is set as the threshold for statistical significance. Two conditional logistic regression models were used to assess the crude and adjusted suicide risk in suicides and controls with regard to different antidepressant classes (Model 1) and compounds (Model 2). Stratified analyses were performed according to adherence and current use of antidepressants. Subjects who were not prescribed the index antidepressant (i.e. SSRI, SNRI, TCA or other) were used as reference for the conditional regression analyses (OR = 1.0).

Descriptive and inferential analyses were conducted using the statistical software Stata/SE (version 13.1).

## Results

The total number of suicides in FVG during the years 2005–2014 was 1260. Suicides treated with antidepressants in the 2 years prior to death were 876. The proportion of suicides treated with antidepressants was greater in females (*N* = 268; 81%) than that in males (*N* = 608; 65%) and that in subjects older than 60 years of age (*N* = 464; 88%) than that in younger (*N* = 412; 56%).

The suicide rate in FVG was 9.4 per 100.000 inhabitants in 2005 and 11.6 per 100.000 inhabitants in 2014. The percentage of suicides treated with antidepressants was 69.5% during the 10-year period.

### Diagnoses and antidepressant prescriptions

Diagnoses registered in cases and controls are summarised in Table 1.

**Table 1:** Numbers (*N*), percentages (%), crude and adjusted odds ratio (OR) and 95% confidence intervals (95% C.I.) of suicide in antidepressant users in the 730 days prior to index date. Data are provided according to antidepressant classes (Model 1) and specific antidepressants (Model 2). Statistically significant ORs and 95% C.I.s are highlighted in bold

	Cases (*n* = 876)		Controls (*n* = 4380)		Crude suicide risk		Adjusted suicide risk^e,f^	
	*N*	%	*N*	%	OR	95% C.I.	OR	95% C.I.
Model 1								
Affective disorders^a^	53	6.0	13	0.3	**23.6**	**12.3**–**45.2**	**10.5**	**5.2**–**21.2**
Non-affective disorders^a^	107	12.2	62	1.4	**9.4**	**6.8**–**13.0**	**7.0**	**4.9**–**10.0**
Somatic disorders^a^	389	44.4	2321	53.0	**0.7**	**0.6**–**0.8**	**0.6**	**0.5**–**0.7**
Switches	139	15.9	537	12.2	**1.4**	**1.1**–**1.7**	1.1	0.9–1.4
Combinations	359	41.0	1283	29.3	**1.8**	**1.5**–**2.1**	**1.4**	**1.1**–**1.7**
SSRI	827^b^	94.4	3987^b^	91.0	**1.7**	**1.2**–**2.3**	**1.6**	**1.1**–**2.2**
SNRI	610^b^	69.6	2788^b^	63.6	**1.3**	**1.1**–**1.6**	1.1	0.9–1.4
TCA	451^b^	51.5	2005^b^	45.8	**1.3**	**1.1**–**1.5**	1.0	0.9–1.3
Other	452^b^	51.6	1912^b^	43.6	**1.4**	**1.2**–**1.6**	1.1	0.9–1.3
Model 2								
Switches	414	47.3	1777	40.6	**1.4**	**1.2**–**1.6**	0.9	0.7–1.2
Combinations	523	59.7	1971	45.0	**2.0**	**1.7**–**2.3**	**1.7**	**1.4**–**2.2**
Paroxetine	623	71.1	2918	66.6	**1.2**	**1.1**–**1.5**	1.0	0.8–1.2
Sertraline	535	61.1	2415	55.4	**1.3**	**1.1**–**1.5**	1.0	0.8–1.2
Citalopram	534	61.0	2355	53.8	**1.4**	**1.2**–**1.6**	1.1	0.9–1.3
Escitalopram	501	57.2	2151	49.1	**1.4**	**1.2**–**1.7**	1.1	0.9–1.4
Fluoxetine	298	34.0	1364	31.1	1.1	1.0–1.3	0.9	0.8–1.1
Fluvoxamine	131	14.9	586	13.4	1.1	0.9–1.4	0.9	0.7–1.1
Venlafaxine	520	59.4	2418	55.2	**1.2**	**1.0**–**1.4**	1.0	0.8–1.2
Duloxetine	354	40.4	1496	34.2	**1.3**	**1.1**–**1.6**	1.1	0.9–1.3
Amitriptiline	328	37.4	1239	32.8	**1.2**	**1.1**–**1.4**	1.0	0.9–1.2
Clomipramine	238	27.2	1084	24.7	1.1	1.0–1.3	0.9	0.7–1.1
Nortriptiline	34	3.9	134	3.1	1.3	0.9–1.9	1.0	0.6–1.6
“Other TCA”^c^	51	5.8	189	4.3	**1.4**	**1.0**–**1.9**	1.0	0.7–1.5
Trazodone	300	34.2	1277	29.2	**1.3**	**1.1**–**1.5**	1.0	0.9–1.3
Mirtazapine	207	23.6	754	17.2	**1.5**	**1.3**–**1.8**	1.2	1.0–1.4
Reboxetine	67	7.6	294	6.7	1.1	0.9–1.5	1.0	0.7–1.3
Bupropion	60	6.8	232	5.3	1.3	1.0–1.8	1.0	0.7–1.3
Mianserin	47	5.4	202	4.6	1.2	0.8–1.6	1.0	0.7–1.4
“Other”^d^	4	0.5	6	0.1	3.3	0.9–11.8	3.7	1.0–13.7

*AD* antidepressants, *SSRI* selective serotonin reuptake inhibitor, *SNRI* serotonergic noradrenergic reuptake inhibitor, *TCA* tricyclics, *Other* “other” antidepressants

^a^Crude and adjusted ORs are shown only in Model 1

^b^The sum is lower than the sum of patients within each specific antidepressant, due to switches and combinations

^c^Trimipramine, imipramine,desipramine, dosulepine, maprotiline

^d^Ademetionin, phenelzine

^e^Adjusted for AD classes, switches to other AD classes, combinations with other AD classes, affective psychiatric disorders, non-affective psychiatric disorders and somatic disorders (Model 1)

^f^Adjusted for switches to other AD, combinations with other AD, affective psychiatric disorders, non-affective psychiatric disorders and somatic disorders (Model 2)

The mean number of prescriptions filled in the 730 days before the index day was 14.2 in cases (median = 9.5; range = 1–138) and 10.5 in controls (median = 6; range = 1–98). SSRI accounted for more than the 90% of the prescriptions in cases, as well as in controls.

### Adherence and current use of antidepressants

Three-hundred thirty-four (38%) cases and 1207 (27%) controls had an MPR ≥ 80%, whilst a current use of antidepressants at the time of the index date was found in 392 cases (45%) and 1382 controls (31%).

Only 235 cases (26.8%) and 797 controls (18.2%) both adhere and currently use antidepressants at the time of the index date.

### Treatment modifications

The number of cases who switched or combined any antidepressant compound at least once during the 730 days prior to the index date was, respectively, 414 (47%) and 523 (60%), whilst controls were respectively 1777 (41%) and 1971 (45%). The number of switches and combinations with regard to antidepressants classes is summarised in Table 1.

Around 20% of cases and controls switched SNRI, TCA and other antidepressants during the study period, whilst 17% of cases and 13% of controls switched SSRI. More than 60% of the cases combined TCA and other antidepressants with other classes, whilst controls were around 50%. Forty-three percent cases and 32% of controls combined SSRI with other classes.

### Antidepressants monotherapy

When only subjects who had neither switched nor combined antidepressant classes were considered, 51 cases out of 491 (10%) and 204 controls out of 2895 (7%) had an MPR ≥ 80% in the 730 days prior to the index date. SSRI had the lowest proportion with regard to MPR ≥ 80% in both cases and controls (8 to 11.5%), whilst TCA and other antidepressants had the highest proportion (16.2 to 19%) (Online Resource 1).

When the current use of antidepressants at the time of the index date was assessed, cases using only one antidepressant class were 134 (27%) and controls were 512 (18%).

### Conditional regression analyses

As summarised in Table 1, in the crude analysis, the risk of suicide was increased in all antidepressant classes (Model 1), as well as in 10 out of 18 antidepressant compounds (Model 2). After adjusting for treatment modifications and for psychiatric and somatic diagnoses, the risk of suicide increased significantly only in SSRI (OR = 1.6), as well as in combinations and in affective and other psychiatric diagnoses (Model 1). In all different antidepressant compounds, the adjusted risk was not significant (Model 2).

**Fig. 1:**
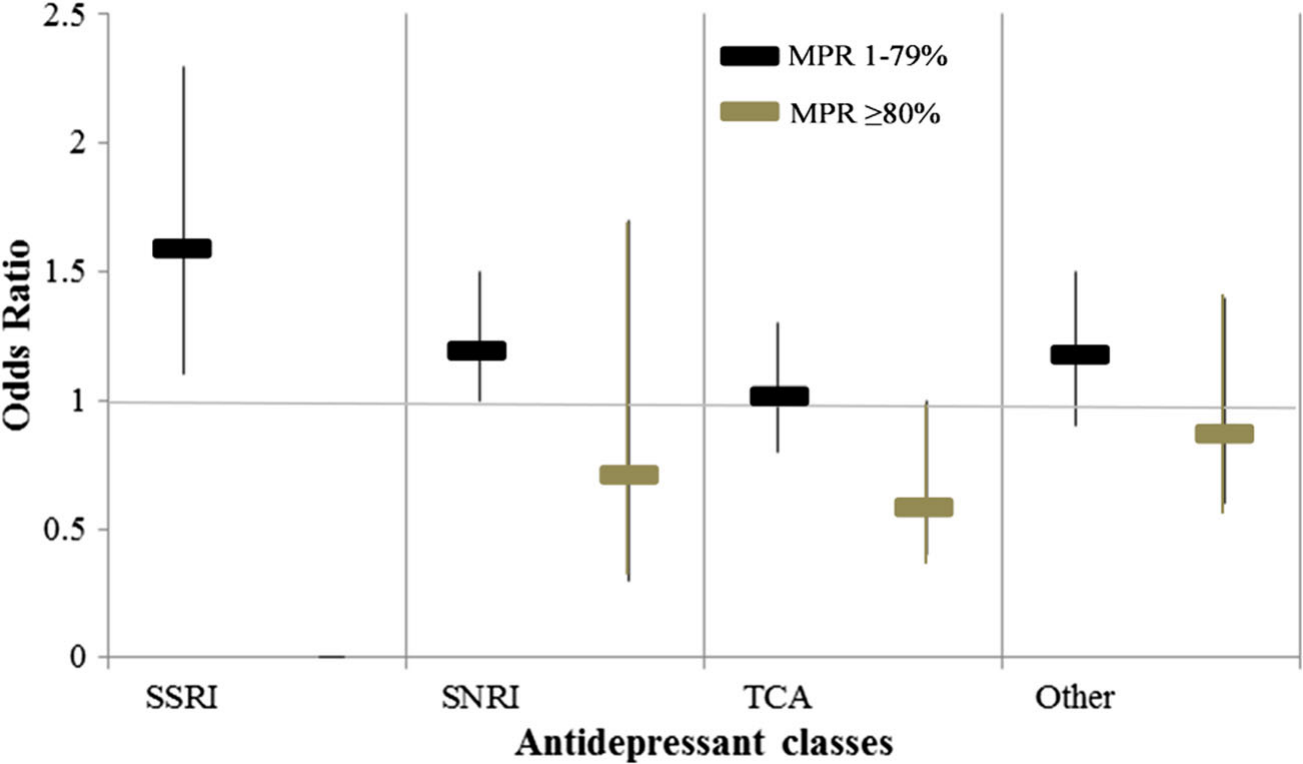
Adjusted odds ratio (OR) and 95% confidence intervals (95% C.I.) of suicide in antidepressant (AD) users according to the adherence to antidepressants in the 730 days prior to index date. The medical possession ratio (MPR) was used to assess adherence to treatment. Data are provided according to AD classes. Stratified analysis was performed comparing subjects who were not adherent to treatment (MPR 1–79%) with subjects adherent to treatment (MPR ≥ 80%). ORs were adjusted for AD classes, switches to other AD classes, combinations with other AD classes, affective psychiatric disorders, non-affective psychiatric disorders and somatic disorders

In the stratified analyses, the risk of suicide decreased in all classes, when comparing subjects with an MPR ≥ 80% to subjects with an MPR 1–79%, but it was significant only in SSRI (OR = 1.6, 95% C.I. = 1.1–2.3) and in SNRI (OR = 1.2, 95% C.I. =1.0–1.5) with an MPR 1–79% (Fig. 1). The risk could not be assessed in subjects adherent to SSRI, since all cases and controls adherent to antidepressants (MPR ≥ 80%) had at least one prescription of SSRI.

**Fig. 2:**
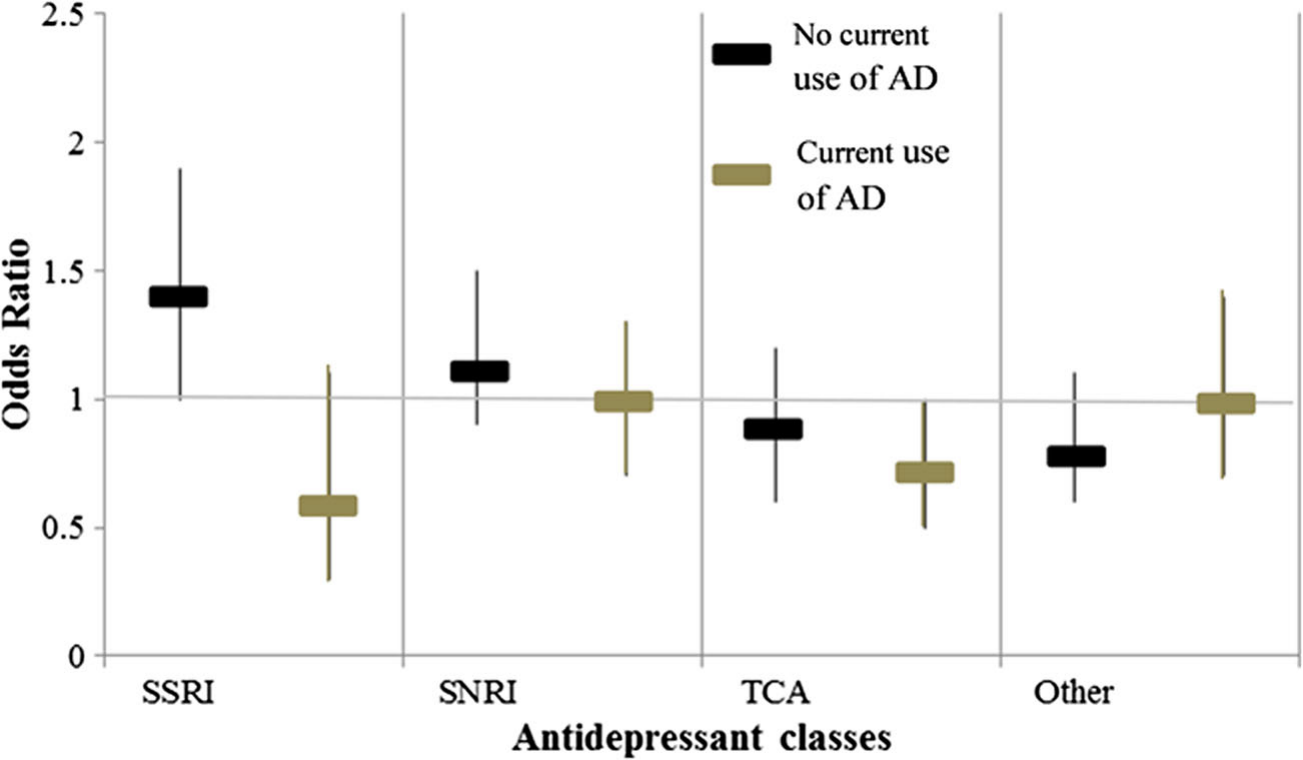
Adjusted odds ratio (OR) and 95% confidence intervals (95% C.I.) of suicide in antidepressant (AD) users according to the current use of antidepressants sufficient to cover the time of the index date. Data are provided according to AD classes. Stratified analysis was performed comparing subjects who were not current users of AD with subjects who were current users. ORs were adjusted for AD classes, switches to other AD classes, combinations with other AD classes, affective psychiatric disorders, non-affective psychiatric disorders and somatic disorders

A decreasing risk was found in SSRI users, when stratified analyses compared subjects that were currently using antidepressants at the time of the index date to those that were not. A higher risk of suicide was significant in subjects who did not currently use SSRI (OR = 1.4, 95% C.I. = 1.0–1.9), whilst current TCA users showed a lower risk (OR = 0.7, 95% C.I. = 0.5–1.0) (Fig. 2).

## Discussion

This study could not assess significant associations between antidepressants and suicide risks, with regard to different antidepressant classes, but SSRI, and all antidepressant compounds. The finding of a higher suicide risk in SSRI was in contrast with a previous case-control study, which did not observe such higher risk [25]. A post-mortem toxicological screening also demonstrated a lower suicide risk in SSRI users [26].

However, we found a decreasing trend in suicide risk when subjects who adhered to antidepressants were compared to subjects who did not adhere. Similar findings were observed when current use of antidepressants was assessed. Although we did not find previous studies assessing the difference in suicide risk with regard to adherence in the general population, Danish studies observed that the risk of suicide decreased along with a continued treatment with antidepressants, assessed by the number of prescriptions [27, 28]. Current use of antidepressants had been further found to be associated to a lower suicidal risk in studies at the population level, with SSRI associated to the lowest suicide risk [20, 21].

In our study, SSRI was the antidepressant class more frequently prescribed, consistent with other Italian and international studies [3, 8, 10, 12, 29], with paroxetine the most prescribed SSRI [10]. We demonstrated, however, that only one in ten subjects prescribed SSRI alone adhered to treatment. A low adherence to antidepressants had been found in several studies among depressed patients [6–11, 14]. Our finding of an adherence rate twice as high in TCA users compared to that of SSRI users, nonetheless, might reflect a greater efficiency of TCA in treatment-resistant depression [27], as well as the fact that patients may be more experienced in using older medications for their illness [7]. This might also explain our finding of a lower suicide risk in subjects currently using TCAs. On the other hand, the higher adherence found in “other antidepressants” such as trazodone and mirtazapine is not easily explainable. It may be confounded by indication, since these antidepressants are widely used in sleeping disorders, with positive outcomes [30].

The high rate of treatment modifications that we observed in suicide victims, as well as the higher suicide risk in subjects who combined antidepressants, is also of interest. Although the indication for switching or combining antidepressants regards the patients with treatment-resistant depression [31], previous studies have suggested that patients who receive more prescriptions [32], as well as frequently change treatment [11, 15, 33], may be more resistant to antidepressants. This can be associated with severe depressive symptoms and a consequent greater suicidal risk [27]. Among the antidepressant classes, SSRIs were less likely to be subjected to treatment modifications, as observed in other research [11, 34]. This may be related to their better tolerability [11]. On the other hand, TCA and “other” antidepressants were combined with other classes twice as often as SSRI, indicating such classes being more used for combination strategies [35].

It should be taken in account that in Italy, the majority of antidepressant prescribers are GPs, who might prefer to prescribe SSRIs, due to their milder side-effect profile. Previous studies, however, observed that GPs may be less able to monitor treatment and to assess and manage suicide risk when compared to psychiatrists [10, 11, 14]. GPs might also be more likely to either stop previous treatment or to modify the treatment frequently in case of resistant depression.

### Strengths and limitations

The strength of this study is that it is a population-based study of all suicides treated with antidepressants occurring during a 10-year period with matched controls. It is adjusted for several confounding factors, such as in-patient diagnoses and treatment modifications. The study design avoids information and selection bias, since it is based on administrative data with full coverage of the regional population [19].

Some limitations should be taken into account. Only antidepressant prescriptions issued by GPs and other public physicians were available from the health database. These, however, constitute 90% of the prescriptions [3].

As in other studies based on prescription registers [7, 11, 14, 18, 19, 27, 36], patients’ actual adherence and compliance to treatment could not be assessed. We used the DDD to assess the MPR, however, one DDD only approximates the average recommended dose per day [36]. Therefore, a possible overestimation of the drug compliance should be taken into account.

Stratified analyses were in some instances hindered by the fact that suicide is a rare event that the available suicide data in this region over 10 years provides limited statistical power. Interesting but not possible analyses were, for example, in subjects with specific age groups or genders or in subjects who used only one class of antidepressant during the study period, the directions of switch or the types of combinations within different antidepressant classes. The database did not provide information on the reasons for treatment changes.

Analyses of diagnoses were only possible for those subjects who had received in-patient care, who probably were more severely ill [19]. The fact that outpatient diagnoses were not available may have led to an underestimate of affective disorders as an indication for antidepressant. This, however, seems a minor issue, since previous database analyses showed that antidepressants are prescribed for depression in more than half of the cases [37]. Furthermore, a previous study indicated that the ability of Italian GPs to detect moderate to severe depression is satisfactory [29].

Other limitations regarded the inability of adjusting the analyses for other factors, such as socio-economic variables, as well as the lack of information on suicide methods, above all antidepressant poisoning [19].

## Conclusions

Notwithstanding the limitations, our observations support the hypothesis that antidepressant treatment is an important factor for preventing suicide. This is supported by the fact that long adherence and current use of antidepressants at the time of death were associated with a lower suicide risk. Since primary care physicians are the most common prescribers of antidepressants, they should ascertain that antidepressants are properly used.

Further research is needed to more closely examine the links between antidepressant use and suicide risk.

## Electronic supplementary material

ESM 1 (DOCX 90 kb).
