# Supplementary material for: Antidepressant use in suicides: a case-control study from the Friuli Venezia Giulia Region, Italy, 2005–2014
Source: Eur J Clin Pharmacol. 2017 Mar 24;73(7):883–90. doi: 10.1007/s00228-017-2236-0 (PMC5486927; doi:10.1007/s00228-017-2236-0)

**Fig 1** Adjusted odds ratio (OR) and 95% confidence intervals (95% C.I.) of suicide in antidepressants (AD) users according to the adherence to antidepressants in the 730 days prior to index date. The medical possession ratio (MPR) was used to assess adherence to treatment. Data are provided according to AD classes. Stratified analysis was performed comparing subjects who were not adherent to treatment (MPR 1-79%) with subjects adherent to treatment (MPR≥80%). ORs were adjusted for AD classes, switches to other AD classes, combinations with other AD classes, affective psychiatric disorders, non-affective psychiatric disorders and somatic disorders.


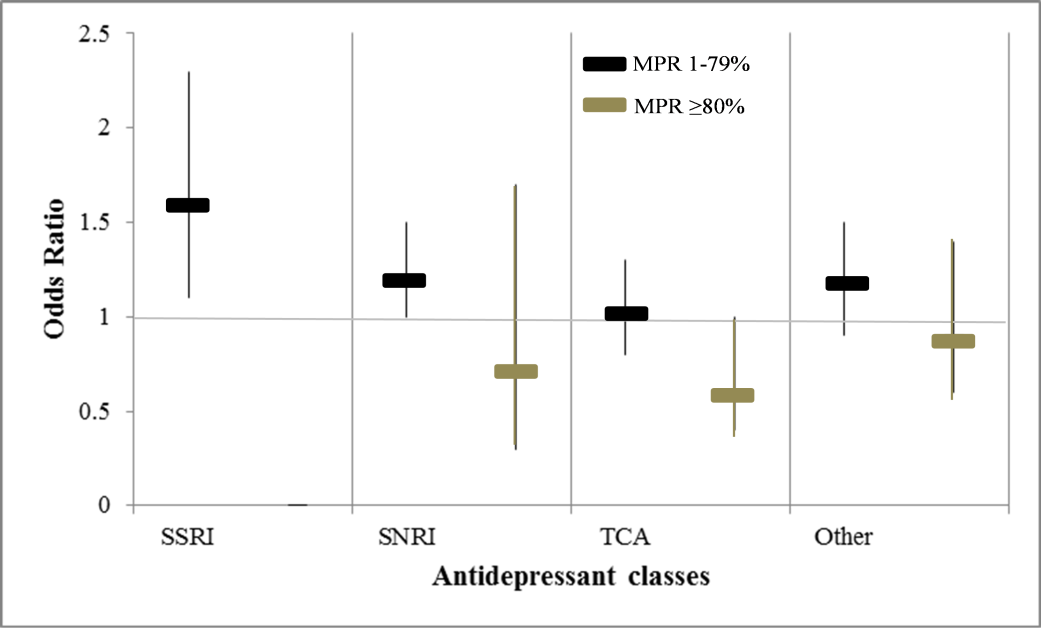


**Fig 2** Adjusted odds ratio (OR) and 95% confidence intervals (95% C.I.) of suicide in antidepressants (AD) users according to the current use of antidepressants sufficient to cover the time of the index date. Data are provided according to AD classes. Stratified analysis was performed comparing subjects who were not current users of AD with subjects who were current users. ORs were adjusted for AD classes, switches to other AD classes, combinations with other AD classes, affective psychiatric disorders, non-affective psychiatric disorders and somatic disorders


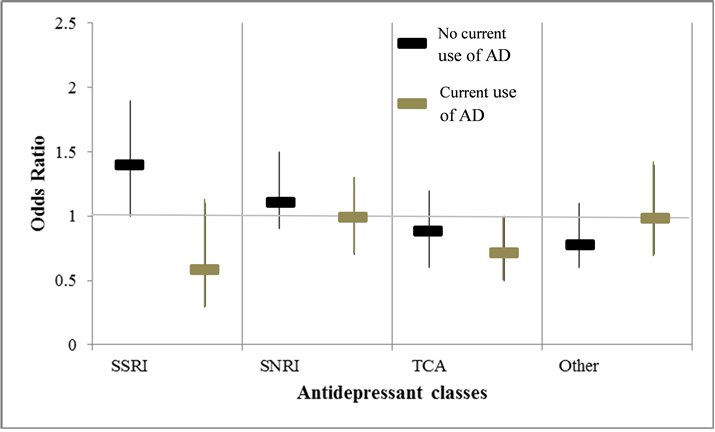

Supplement: Supplementary file 1 — (DOCX 90 kb) [file 228_2017_2236_MOESM1_ESM.docx]
